# Endoscopic variceal ligation-induced ulcer bleeding: incidence by indication, real-world treatment, and outcomes

**DOI:** 10.1186/s12876-026-05141-5

**Published:** 2026-07-24

**Authors:** Thomas Vasilakis, Simon Schauer, Donata Grajecki, Alexander Wree, Christian Jürgensen, Frank Tacke, Cornelius Engelmann

**Affiliations:** 1 Department of Hepatology & Gastroenterology, Charité – Universitätsmedizin Berlin, Charité Campus Mitte, Charitéplatz 1, Berlin, 10117 Germany; 2 Department of Hepatology & Gastroenterology, Charité – Universitätsmedizin Berlin, Charité Virchow Klinikum, Augustenburger Platz 1, Berlin, 13353 Germany; 3 Department of Internal Medicine A, Fleischmannstrasse 8, Greifswald, 17489 Germany; 4 Department of Gastroenterology, Vivantes Klinikum Friedrichshain, Landsberger Allee 40, Berlin, 10249 Germany

**Keywords:** Bleeding, Ligation, Varices, Liver cirrhosis, Post-banding ulcer

## Abstract

**Purpose:**

Endoscopic variceal ligation (EVL) - induced ulcer bleeding is a rare but potentially life-threatening complication. While the incidence following elective and emergency EVL has been reported, semi-elective procedures performed during hospitalization for acute decompensation of cirrhosis have not been separately examined. Additionally, real-world data on endoscopic treatment strategies, hemostasis rates and outcomes are scarce.

**Methods:**

We conducted a retrospective cohort study analyzing all ligation procedures performed in adults with portal hypertension and esophageal varices at Charité University Hospital, Campus Virchow and Campus Mitte, from 01/01/2016 until 06/30/2023. We assessed incidence and risk factors by ligation indication, described endoscopic treatment strategies and analyzed predictors of 5-day rebleeding and 6-week mortality.

**Results:**

Among 1,864 EVLs, 61 (3.3%) resulted in EVL-induced ulcer bleeding; 60 were analyzed. Incidence varied significantly by indication: 0.44% after elective, 8.5% after emergency and 15.9% after semi-elective EVL. Repeat ligation was the most common endoscopic treatment (32.4%), followed by fibrin glue (14.7%) and balloon tamponade (11.7%); the overall primary hemostasis rate was 82.8%. Ten patients (16.7%) underwent TIPS. The 5-day rebleeding rate was 25% and the 6-week mortality rate 41.7%. Multivariate logistic regression analysis revealed that 5-day rebleeding (OR: 8.05; 1.66–39.2; *p* = 0.01) and post-bleeding sepsis (OR 7.27; 1.15–45.73; *p* = 0.035) were strongly associated with 6-week mortality.

**Conclusions:**

Semi-elective EVL carries 45-fold higher odds for EVL-induced ulcer bleeding. Endoscopic hemostasis is achievable, but early rebleeding and post-bleeding sepsis drive mortality, suggesting that considering early TIPS in high-risk patients and preventing sepsis deserve more attention.

**Supplementary Information:**

The online version contains supplementary material available at 10.1186/s12876-026-05141-5.

## Introduction

Gastroesophageal varices are one of the most common complications of liver cirrhosis and portal hypertension. The prevalence of gastroesophageal varices increases up to 85% as the disease progresses [1]. Endoscopic variceal ligation (EVL) is recommended as a primary and secondary prophylaxis for esophageal variceal bleeding and is the first-line treatment for acute esophageal variceal bleeding [2, 3]. EVL is safe with few treatment-related complications, such as dysphagia, post-ligation pain, esophageal stricture formation and EVL-induced ulcer bleeding [4].

The ligation procedure causes a tight compression of the varix with vascular compromise, leading to thrombosis, necrosis, formation of an ulcer, and sloughing. The rubber bands slip off approximately 3 to 7 days after the banding, and esophageal ulcerations develop, which usually heal within 2 to 3 weeks [5]. Early slippage of the band leads to an incomplete occlusion of the varix with an immature thrombus, which may lead to bleeding.

The incidence of EVL-induced ulcer bleeding is low (2%–8%) [6–8], but it has a high mortality rate (11–63%) [6, 7, 9–11]. Several studies have assessed the risk factors associated with the occurrence of this complication, yielding inconsistent results. These discrepancies likely reflect heterogeneity introduced by pooling of procedures across indications without accounting for differences in baseline disease severity. Nevertheless, a recent meta-analysis observed that a higher MELD (Model for End-stage Liver Disease) score, emergency ligation and Child-Pugh Stadium C were repeatedly identified as risk factors [10, 12].

Currently, there is no specific guideline for the endoscopic treatment of EVL-induced ulcer bleeding. Published data show that most endoscopists perform repeat ligation first [12]. However, band ligation is often technically challenging due to scarring after previous ligation. In these cases, alternative treatments have been described in case reports and small series including injection of epinephrine [6], cyanoacrylate [13] or fibrin glue [6], through the scope [6, 14] or over the scope clip [15], topical hemostatic agents [16], self-expandable metal stent [17], balloon tamponade [6, 18], argon plasma coagulation [18] and transjugular intrahepatic portosystemic shunt (TIPS) [9]. Medical treatment with proton pump inhibitors (PPI) ± octreotide is a possible strategy in cases of no active bleeding [18].

Despite the clinical significance of this complication, important aspects of EVL-induced ulcer bleeding remain inadequately characterized. The reported incidence of 2–8% pools elective, semi-elective, and emergency procedures, obscuring potentially important differences in bleeding risk across clinical contexts. In particular, semi-elective EVL, performed during hospitalization for acute decompensation of cirrhosis, has not been yet separately examined, although it may represent a higher risk setting in comparison to elective EVL. Furthermore, available data on hemostasis derive exclusively from small case series reporting rates of 45–80%, without stratification by individual endoscopic treatment modality [6, 19]. Beyond hemostasis, data on clinical outcomes including 5-day rebleeding rates and independent predictors of mortality remain largely unreported [6, 12].

Therefore, our aims were to assess EVL-induced ulcer bleeding incidence stratified by ligation indication, to describe real-world endoscopic treatment strategies and achievable hemostasis rates and to analyze independent predictors of 5-day rebleeding and 6-week mortality.

## Methods

We conducted a retrospective cohort study by reviewing the medical records of adult patients with liver cirrhosis/portal hypertension who received EVL at the Charité-Universitätsmedizin Berlin, Campus Virchow-Klinikum and Campus Charité Mitte, in Berlin from January 1st, 2016, until June 30th, 2023. The EVLs were performed as primary or secondary prophylaxis for variceal bleeding either as an elective procedure or semi-elective in cases of inpatients with acute decompensation of cirrhosis (new or worsening of ascites and/or new or worsening hepatic encephalopathy and/or hepatorenal syndrome and/or non-variceal gastrointestinal bleeding). Emergency EVLs for the treatment of acute esophageal variceal bleeding were also included. If the EVL responsible for the ulcer bleeding episode had been performed at another institution (*n* = 14), those patients were excluded to minimize procedural confounding. This exclusion may have introduced selection bias towards less severe presentations, as transferred patients may represent a higher-risk population. We calculated overall EVL-induced ulcer bleeding rates and stratified them by EVL indication (elective, semi-elective, emergency) to assess risk variation across clinical settings.

### Case-control sub-study

In this sub-study we compared our bleeding cohort with controls that received EVL but developed no EVL-induced ulcer bleeding during the same period, in order to identify risk factors for the occurrence of this complication. We randomly selected our controls (computer-generated) with a case/control ratio of 1:1.85. This ratio was calculated based on the size of our cohort group, the detection of differences of medium effect size (0.41), and the standard level of significance and power (5% and 80%, respectively). We matched our controls in terms of year of EVL and setting of ligations. Patients that died shortly after EVL, such as patients in a palliative oncological setting, were excluded (*n* = 3).

### Endoscopy

EVL was performed by using the six shooter Saeed multiband ligator (Cook Medical Endoscopy, Limerick, Ireland), maximum 6 ligations per session were applied. In total, 15 endoscopists with different levels of expertise performed the EVLs at the two endoscopy sites; a gastroenterology consultant supervised trainees. We diagnosed EVL-induced bleeding when detecting active bleeding or stigmata of previous bleeding in form of blood clots or blood residues on the ulcers in endoscopy. In the case of EVL-induced ulcers with a clean fibrin-covered base, the diagnosis of EVL-induced ulcer bleeding was made, provided that no other possible bleeding causes were detected. We defined this endoscopy as index endoscopy. Based on the proposed classification of Jamwal et al. [9], we described the EVL-induced ulcer bleeding as follows: spurting bleeding, oozing bleeding, ulcer with non-bleeding visible vessel, ulcer with adherent clot, ulcer with hematin-covered base, and ulcer with fibrin-covered base. Given the limited number of cases in the non-bleeding visible vessel, adherent clot, and hematin-covered base categories, findings were grouped into active versus non-active bleeding for comparative analyses. Treatment selection was non-randomized and the treatment approach was influenced by bleeding severity, hemodynamic stability, endoscopists´ assessment and expertise. Therefore, direct treatment comparisons are not appropriate due to confounding by indication. Technical success was defined as successful deployment of intended endoscopic therapy and primary hemostasis as cessation of active bleeding during index endoscopy. Primary hemostasis in cases of balloon tamponade was documented based on hemodynamic stability of the patient after the endoscopy and no signs of active bleeding in the second look endoscopy the following day, given that immediate post-intervention endoscopic assessment is not feasible during balloon tamponade. No standardized antibiotic prophylaxis protocol was applied; administration was left to clinical judgment on an individual basis.

### Clinical outcomes

Clinical outcomes comprised 5-day rebleeding rate and 6-week all-cause mortality counted after the EVL-induced bleeding episode. We also identified risk factors for these outcomes and assessed the cause of death. We chose 5-day rebleeding and 6-week mortality based on the current recommendations of the latest BAVENO VII consensus for studies on treating acute variceal bleeding [3]. Acute on chronic liver failure (ACLF) was diagnosed according to the latest EASL Guidelines [20]. Along MELD score disease severity was assessed using the validated Chronic Liver Failure-Consortium (CLIF-C) scores [21]. The CLIF-C acute decompensation (CLIF-C AD) score was calculated in all patients with acute liver decompensation. The CLIF-C organ failure (CLIF-C OF) score was calculated, when organ failure in any of the following six organ systems, liver, kidney, brain, coagulation, circulation, and respiration was present. The CLIF-C ACLF score was applied in patients meeting ACLF criteria. We defined sepsis according to the Third International Consensus Definitions (Sepsis-3): documented or suspected infection with an acute increase in sequential organ failure assessment (SOFA) score of two [22]. Post-bleeding sepsis was specifically defined as new-onset sepsis developing within five days after EVL-induced ulcer bleeding. The five-day time frame was selected to capture infections attributable to bleeding-related mechanisms (aspiration, bacterial translocation, and procedure-related complications), while minimizing attribution of unrelated hospital-acquired infections. This is consistent with prior data demonstrating a median time to new-onset infection of 3–5 days after variceal bleeding [23].

### Statistical analysis

We performed the statistical analysis using SPSS 29.0 (SPSS, Inc., IBM, Chicago, IL). We reported continuous variables as median with interquartile range and compared them using non-parametric tests as appropriate. We reported categorical variables as frequencies with percentages and compared using the chi-squared or Fisher´s exact test when events where ≤ 5. A p-value < 0.05 indicated statistical significance. Given the potential confounding by indication, we presented treatment effectiveness descriptively without formal statistical comparisons. We used binary logistic regression models to investigate the risk factors associated with 5-day rebleeding and 6-week mortality. Given the absence of dedicated prior literature on mortality predictors in EVL-induced ulcer bleeding, the univariate analysis was exploratory. However, certain variables were pre-selected based on biological plausibility and evidence from the broader variceal bleeding literature, namely post-bleeding sepsis, 5-day rebleeding, active bleeding at endoscopy, and ACLF [3]. Variables with a p-value of ≤ 0.05 in univariate analysis were considered for multivariate models using the “one variable per 10 events” rule to avoid overfitting [24]. We used goodness-of-fit statistics (Akaike-Information Criterion) to identify the most robust model [24]. We also performed a Cox proportional hazards regression for the parameters of our logistic regression model as a sensitivity analysis to account for the timing of events. We excluded variables that had more than 5% missing data (albumin levels 6.7%, Child-Pugh classification 6.7%, fibrinogen levels 25%, prior history of variceal bleeding 26.7%, prior ligation 15%, prior hospitalization within six months 23.3%). When assessing variables with missing data, we performed a listwise deletion of cases. Due to the low proportion of missing data for the remaining variables the likely impact of this approach on the overall findings was considered minimal.

## Results

### Cohort characteristics

During the study period, 975 patients underwent 1,864 EVLs, among which 61 cases (3.3%) with EVL-induced ulcer bleeding occurred. After excluding one case due to a missing endoscopy report, we analyzed 60 cases. Each case occurred in a unique patient, ensuring independence of observations. Table 1 summarizes the characteristics of the cohort, including epidemiological data, baseline medications, variceal status and laboratory parameters at baseline EVL.

**Table 1:** Patient characteristics at baseline EVL in the bleeding cohort

Parameters		
Age, median (IQR)	56.5	46–68
Male sex, n (%)	47	78.3
Etiology of Portal Hypertension	**N**	**%**
PVT	5	8.3
Hepatic causes	53	85.0
- ALD	30	50.0
- Viral hepatitis	14	23.3
- MASLD	7	11.7
- Others	15	25.0
Post-hepatic causes	2	3.3
Clinical Status at Baseline	**N**	**%**
HCC	14	23.3
TIPS	3	5.0
Dialysis	2	3.3
ACLF		
- No ACLF	43	71.7
- ACLF 1	9	15.0
- ACLF 2	5	8.3
- ACLF 3	3	5.0
Hepatic encephalopathy present	19	31.6
Ascites present	53	88.4
Medications at Baseline	**N**	**%**
Non-selective b-blockers	26	43.3
Proton pump inhibitors	39	65.0
Anticoagulants	12	20.3
Endoscopy at Baseline	**N**	**%**
III + IV Grade varices	26	43.3
Red spots on varices	38	63.3
Number of ligations, median (IQR)	5	4–6
Laboratory Values at Baseline	**Median**	**IQR**
Bilirubin [mg/dl]	2.8	1.3–6.1
Creatinine [mg/dl]	1.1	0.82–1.61
MELD score	17.5	13–22
Platelets [/nl]	108.5	67.3–175.8
ALT [U/l]	36	24–62.5
AST [U/l]	63	50–93
Hb [g/dl]	9.3	8.1–10.8
INR	1.44	1.24–1.71
aPTT [sec]	43.7	36.9–50.0
CLIF-C AD score*	55	48–62
CLIF-C OF score*	9	8–13
CLIF-C ACLF score*	56	47–61

Continuous data are shown as median and interquartile range, while categorical data are shown as absolute and relative frequencies

A detailed comparison of baseline characteristics is available at the online resource

*ALD* Alcohol-associated liver disease, *MASLD* Metabolic dysfunction-associated steatotic liver disease, *PVT* portal vein thrombosis, *aPTT* activated partial thromboplastin time, *HCC* hepatocellular carcinoma, *TIPS* trasjugular intrahepatic portosystemic shunt, *ACLF* acute on chronic liver failure, *MELD* Model for End-stage Liver Disease, *IQR* interquartile range

*CLIF-AD Score was not documented in cases of elective EVL. CLIF-OF and CLIF ACLF were documented only in cases of ACLF. Missing data: 1.7% for medications at baseline

### Indication of EVL

**Fig. 1:**
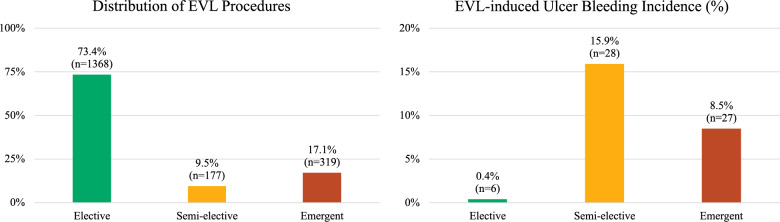
Procedure distribution and EVL-UB incidence based on indication. EVL-UB: endoscopic variceal ligation induced ulcer bleeding

The incidence varied significantly by EVL indication (Fig. 1). The baseline EVLs (*n* = 1,864) were 73.4% elective, 9.5% semi-elective, and 17.1% emergent. The incidence rate of EVL-induced ulcer bleeding was 0.44% after elective, 8.5% after emergent and 15.9% after semi-elective procedures. In comparison to elective EVL a 45-fold increase in odds for EVL-induced ulcer bleeding was observed after semi-elective EVL (OR: 45.5; 95% CI 18.2–111.1; *p* < 0.0001) and a 22-fold increase after emergent EVL (OR: 21.7; 95% CI 9.0–52.6; *p* < 0.0001). The odds for this complication were two times higher after semi-elective EVL in comparison to emergent EVL (OR: 2.03; 95% CI 1.2–3.5; *p* = 0.01). EVL-induced ulcer bleeding occurred at a median of 10 days after EVL. This complication tended to occur earlier after an emergency EVL (8 days; 4–13) in comparison to a semi-elective (11 days; 6–17) or elective EVL (16 days; 11–20), but this result did not reach statistical significance (*p* = 0.15).

### Risk factors for EVL-induced bleeding after semi-elective EVL

Within the semi-elective subgroup, ICU treatment at the time of EVL was significantly more frequent among bleeding patients than controls (14.3% vs. 0%; *p* = 0.01). MELD score and aPTT showed non-significant trends toward higher values in the bleeding group (19.5 vs. 16; *p* = 0.097 and 43.7 vs. 42.3 s; *p* = 0.07, respectively). The time interval between hospital admission and EVL did not differ significantly between groups (4 vs. 5 days; *p* = 0.28). The CLIF-C (AD, OF, ACLF) scores and FIB4 score also did not differ significantly between the two groups and are presented in the online resource.

### Treatment of EVL-induced ulcer bleeding

#### Time to endoscopy

Among patients with EVL-induced ulcer bleeding, 80% (*n* = 48) underwent endoscopy within six hours of clinical presentation. Despite early intervention, 6-week mortality in this group was 50%. The remaining 20% were examined between 6 and 24 h (*n* = 10, 16.7%) or after 24 h (*n* = 2, 3.3%), with a 6-week mortality of only 10%. This counterintuitive finding should not be understood as evidence supporting delayed endoscopy, as it reflects confounding by clinical severity. Patients examined within six hours were hemodynamically unstable; 81.3% required endoscopy in the ICU setting and 60.4% had active bleeding at the time of intervention. In contrast, all patients examined after six hours were clinically stable, underwent endoscopy in the endoscopy unit, and only 16.7% had active bleeding at the time of endoscopy (*p* = 0.034).

#### Endoscopic findings of EVL-induced ulcer bleeding

The EVL-induced ulcer bleeding presented at index endoscopy as follows: 11.7% spurting bleeding, 40% oozing bleeding, 1.7% ulcer with non-bleeding visible vessel, 6.7% ulcer with adherent clot, 5% ulcer with hematin-covered base and 35% ulcer with fibrin-covered base. Active bleeding at the time of endoscopy did not affect 5-day rebleeding (25.9% vs. 22.2%; *p* = 0.75), but it was associated with higher 6-week mortality (68% vs. 40%; *p* = 0.03).

#### Endoscopic therapy of EVL-induced ulcer bleeding

Among the 60 patients, 69 sequential endoscopic interventions were performed with nine patients receiving two treatment modalities. Due to tissue scarring induced by previous ligations, EVL was technically not possible in three cases, either due to the inability to capture the ulcer or immediate slippage of the band. Additionally, hemostasis was not achieved twice, despite successful ligation deployment. In these 5 cases, the endoscopists either proceeded with fibrin glue injection (*n* = 1) or balloon tamponade (*n* = 3) or no further attempt (*n* = 1) in one case of inactive bleeding. Fibrin glue injection was technically feasible in all cases and achieved hemostasis in all but one instance, in which the endoscopist subsequently performed an EVL. Balloon tamponade was also necessary as a salvage treatment in two cases following failed deployment of a self-expanding metal stent (SEMS). One treatment attempt with cyanoacrylate was unsuccessful; in this case hemostasis was achieved with fibrin glue.

**Fig. 2:**
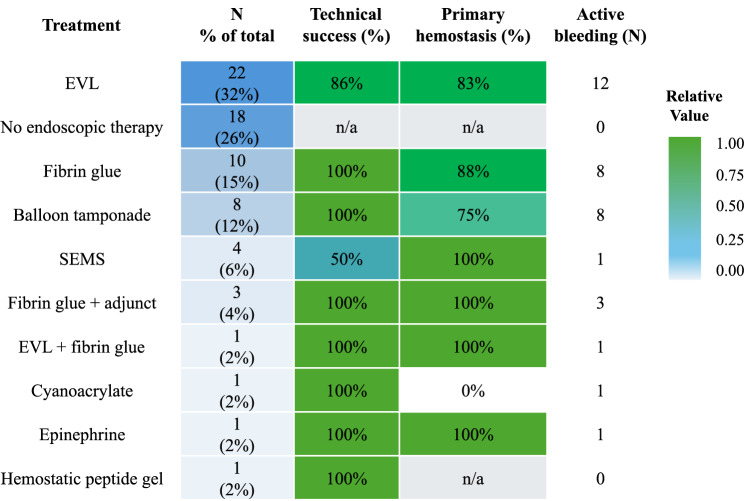
Endoscopic therapies for EVL-UB. Description of all attempted endoscopic therapies used to treat EVL-induced ulcer bleeds. We calculated the technical success rate for all cases, while the primary hemostasis rate was measured only in cases with active bleeding. Some patients received sequential treatments after initial treatment failure; this table shows all attempted interventions (five were not technically successful and four did not achieve primary hemostasis, therefore *n* = 69. Final treatments after excluding unsuccessful attempts are shown in Fig. 3, Fibrin + adjunct: Fibrin glue + epinephrine (*n* = 1), fibrin glue, epinephrine and hemostatic peptide gel (*n* = 1), and fibrin glue with cyanoacrylate (*n* = 1). EVL-UB: endoscopic variceal ligation induced ulcer bleeding

In total, repeat EVL (*n* = 22, 32.4%) was the most commonly used endoscopic treatment, with a technical success rate of 86.4% and primary hemostasis rate of 83.3%. Fibrin glue was the second most common endoscopic modality (*n* = 10, 14.7%), achieving 100% technical success rate and 87.5% primary hemostasis success rate. Balloon tamponade was technically successful in all cases and reached a 75% primary hemostasis rate. Self-expanding metal stents were used in four patients, with successful deployment in two; one stent was removed seven days later following TIPS placement, while the other patient died within 48 h. In one patient, a combination of EVL and fibrin glue was applied, and in three patients, fibrin glue with additional agents were injected (Fig. 2).

Of the eight patients who received balloon tamponade, seven underwent a second-look endoscopy the following day (one patient died prior). Three patients were treated with a metal stent due to either esophageal perforation or deep laceration, one underwent ligation due to rebleeding, and three required no further endoscopic intervention, with two of them having received TIPS prior to balloon removal.

Treatment selection appeared influenced by bleeding severity and clinical context. Among patients with spurting bleeding (*n* = 7), 57% received repeat EVL, while 29% required immediate balloon tamponade. Among those with oozing bleeding (*n* = 24), 25% received repeat EVL, 38% received fibrin-based therapies, and 25% required balloon tamponade.

#### 5-day rebleeding rate

The median time for rebleeding was 2 days (0.5–3.5 days) after the initial ulcer bleeding. Six patients died within 2 days after initial bleeding with no signs of rebleeding; therefore, we excluded them from this analysis. Our cohort’s 5-day rebleeding rate reached 25% (*n* = 13). Rebleeding in total was 26.9% (*n* = 14). Figure 2 illustrates the 5-day rebleeding rate based on the initial endoscopic presentation, and based on the treatment used. We identified hepatic encephalopathy at the time of EVL-induced bleeding as a risk factor for rebleeding (41.2% vs. 6.7%, *p* = 0.002; OR: 2.5, 95% CI 1.2–4.9, *p* = 0.01). Patients with ACLF at the time of the ulcer bleeding tended to have higher 5-day rebleeding rate (OR 3.86, 95% CI 0.92–16.11; *p* = 0.06). Furthermore, rebleeding risk increased significantly with ACLF severity, with 5-day rebleeding rates of 13.3%, 57.1%, and 57.1% for ACLF grades 1, 2, and 3, respectively (*p* = 0.04). MELD Score, CLIF-AD, CLIF-OF, CLIF-ACLF, aPTT, the presence of ascites or portal vein thrombosis, and treatment with PPIs did not play a role in the recurrence of EVL-induced bleeding in our cohort.

#### Management of fibrin-covered ulcers after EVL-induced bleeding

Endoscopic therapy was employed in 7 out of 21 patients (33.3%) with fibrin-covered ulcers (Fig. 2). Patients receiving endoscopic treatment for these ulcers had numerically a higher 5-day rebleeding rate (33.3% vs. 7.1%, *p* = 0.2) and 6-week mortality rate (42.8% vs. 21.4%, *p* = 0.35), but these differences did not reach statistical significance. The presence of ACLF may have influenced the 5-day rebleeding and 6-week mortality rates in this subgroup. Patients with clear fibrin-covered ulcers who received no endoscopic treatment and had no ACLF (*n* = 7), had no rebleeding and 0% 6-week mortality. In contrast, patients who received no endoscopic treatment but had ACLF (*n* = 7) experienced 16.7% rebleeding (1/6 evaluable patients) and 42.9% mortality (3/7), despite identical endoscopic presentation and management approach. The latter comparison did not reach statistical significance (*p* = 0.19), potentially due to small subgroup size. These findings suggest that conservative management may be appropriate for fibrin-covered EVL-induced ulcers in patients without ACLF, as endoscopic intervention did not improve outcomes in this subgroup. Patients managed conservatively tended to have lower MELD scores both at baseline EVL (14; 8–22) and at the time of EVL-induced ulcer bleeding (16.5; 12–28) compared to those who received endoscopic therapy (18;13–22 and 22;16–28, respectively), though this difference did not reach statistical significance (*p* = 0.07).

**Fig. 3:**
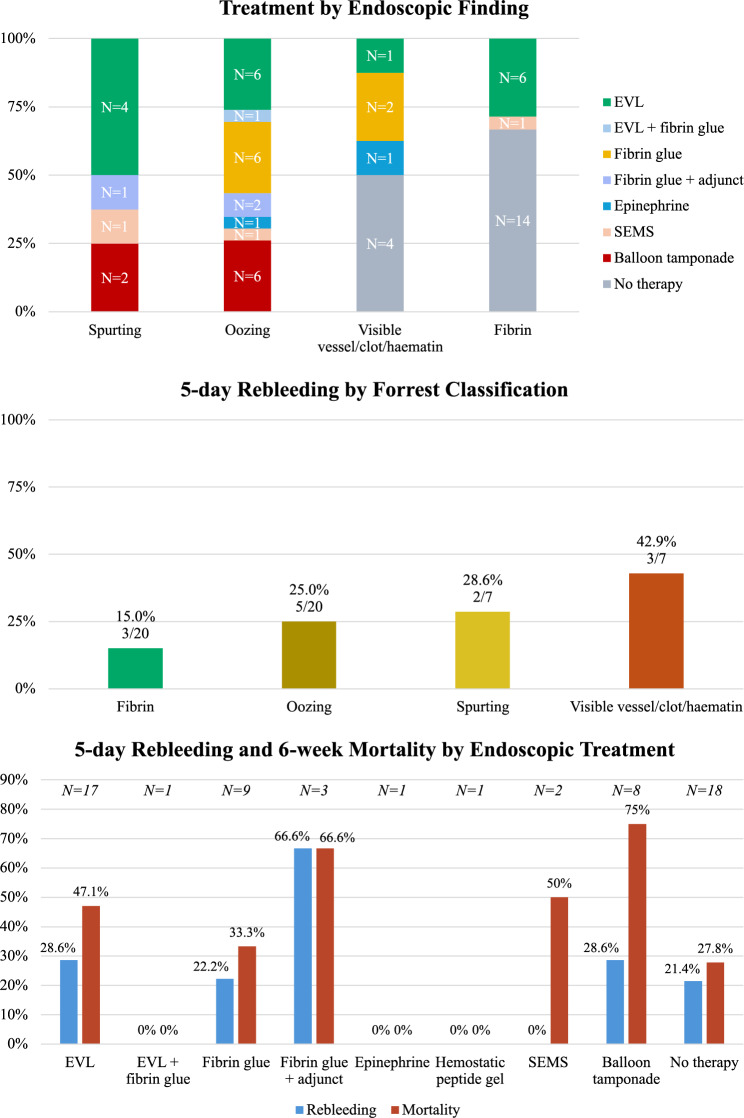
Endoscopic findings, treatment and outcomes of EVL-UB. The first graph illustrates the activity of the bleeding at index endoscopy and the respective treatment that was applied. The second graph shows the 5-day rebleeding rate based on endoscopic findings at index endoscopy (the absolute number and percentage is depicted). Patients that died within 2 days of the ulcer bleeding (*n* = 6) were excluded from this analysis. The third graph shows the 5-day rebleeding rate and 6-week mortality rate based on the endoscopic treatment. EVL-UB: endoscopic variceal ligation induced ulcer bleeding, SEMS: self-expandable metal stent

#### TIPS placement

Ten patients (16.7%) underwent TIPS placement at a median of 2 days (1.5–7.5) after the bleeding episode. The initial endoscopic therapy varied (EVL = 2, fibrin glue = 2, balloon tamponade = 3, SEMS = 1, no treatment = 2). Only two patients received TIPS preemptively, within 72 h after the EVL-induced ulcer bleed and before bleeding recurrence. Despite the early placement, both patients died. Patients who underwent TIPS had more severe liver disease (MELD-Na 27 vs. 21, *p* = 0.034) and almost all developed an infection after EVL-induced bleeding (90% vs. 32%, *p* < 0.001), with post-bleeding sepsis occurring in 70% vs. 10% (*p* < 0.001). The 6-week mortality in TIPS patients was 90%. Whether high mortality reflects disease severity, delayed TIPS timing, or infectious complications cannot be determined from these observational data. No patient received liver transplantation.

### Mortality

#### 6-week mortality rate, in-hospital mortality rate, 30-days mortality

Overall, 6-week mortality rate was 41.7% and in-hospital mortality rate was 46.7%. Three more patients (9.4%), who initially survived the EVL-UB and were discharged, died 30 days after discharge. Six-week mortality varied by EVL indication: 50% after semi-elective 37.5% after emergency and 20% after elective EVL procedures. There was no statistical difference based on indication for EVL. Median time to death was 9 days (IQR 2–21). Most common cause of death was acute on chronic liver failure (52%) followed by uncontrolled infection (28%) (as shown in online resource).

#### Predictors of 6-week mortality after EVL-induced ulcer bleeding

**Fig. 4:**
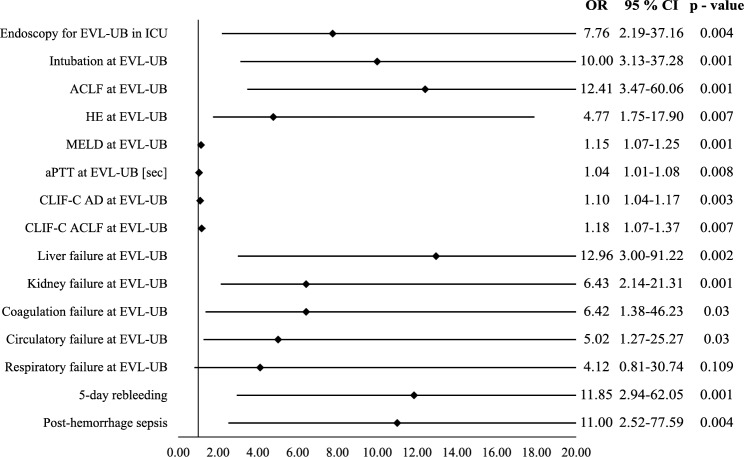
Predictors of 6-week mortality after EVL-UB, univariate analysis. We demonstrate the odds ratios using univariate logistic regression analysis and the respective significance. For brevity purposes, we omitted the variables that had no statistical significance (age, sex, cause of portal hypertension, presence of HCC, any medication at baseline, indication for ligation, creatinine, Hb, INR, post-bleeding infection). The OR regarding brain failure reached infinity because all patients with this complication died, therefore it is not depicted on the figure. The small sample size and limited number of outcome events resulted in wide confidence intervals, reflecting considerable statistical uncertainty in the categorical estimates. EVL-UB: endoscopic variceal ligation induced ulcer bleeding, ACLF: acute on chronic liver failure, HE: hepatic encephalopathy, MELD: model of end stage liver disease, aPTT: activated partial thromboplastin time CLIF: chronic liver failure, AD: acute decompensation

The univariate logistic regression revealed liver failure, 5-day rebleeding, post-bleeding sepsis and ACLF as the strongest predictors for 6-week mortality (Fig. 4). The 6-week mortality rate increased with the severity of ACLF at the time of the EVL-induced bleeding; 33.3% for grade 1, 75% for grade 2 and 91.7% for grade 3. In-hospital mortality of patients with ACLF Grade 3 was 100%. We also observed a 100% 6-week mortality when CLIF-C ACLF > 67. Pneumonia was the most common post-bleeding infectious complication in our cohort, occurring in 26.7% of patients. All cases of post-bleeding pneumonia were either hospital acquired or associated with aspiration or ventilation. Among patients intubated before endoscopy 44.8% (13/29) developed pneumonia, compared to only 13.3% (4/30) of non-intubated patients. No pneumonia occurred in the single patient that required intubation after endoscopy (Table 2). It is important to mention that 18.3% of the patients developed sepsis before the EVL-UB, which was also associated with increased 6-week mortality.

**Table 2:** Risk factors for 6-week mortality after EVL-induced ulcer bleeding

Parameters	Survival (*n* = 35)		Death (*n* = 25)		*P*
Age, median (IQR)	57	45.5–65	56	51–70	0.68
Male sex, n (%)	26	74.3	21	84.0	0.52
Baseline					
Indication for Ligation					0.36
- Elective	4	11.4	1	4.0	
- Semi-elective	14	40.0	14	56.0	
- Emergent	17	48.6	10	40.0	
ICU at baseline	5	14.3	11	44.0	0.014*
Index Endoscopy					
Endoscopy performed in ICU	17	48.6	22	88.0	0.02*
Active bleeding	14	40.0	17	68.0	0.03*
Intubation necessary	10	28.6	19	76.0	< 0.001*
Clinical Status at Index Endoscopy					
ACLF	13	37.1	21	84.0	< 0.001*
- ACLF 1	10	28.6	5	20.0	
- ACLF 2	2	5.7	6	24.0	
- ACLF 3	1	2.9	10	40.0	
Hepatic encephalopathy present	11	31.4	19	76.0	0.01*
Infection	7	20.0	9	36.0	0.17
Sepsis	3	8.6	8	32.0	0.04*
Non-endoscopic Therapy					
PPI before endoscopy	26	74.3	22	88.0	0.37
− 80 mg	9	25.7	15	60.0	0.02*
PPI after endoscopy	33	94.3	25	100	0.51
− 80 mg	21	60.0	22	88.0	0.067
Norepinephrine	11	31.4	21	84.0	< 0.001*
TIPS placement	2	5.7	8	32.0	0.01*
Laboratory values at Index Endoscopy	**Median**	**IQR**	**Median**	**IQR**	**P**_**1**_
Bilirubin [mg/dl]	2.49	0.89–3.87	9.54	1.5–20.2	0.44
Creatinine [mg/dl]	1.24	0.8–1.86	1.41	1.18–2.1	0.48
MELD	17	12–22	28	20.5–35.5	0.44
MELD Na	18	11–24	29	21.5–35	0.32
Platelets [/nl]	120	79–203	87	48.5–122.5	*0.09*
Hb [g/dl]	8.8	7.5–10.3	7.8	7.4–9.1	0.59
INR	1.41	1.15–1.87	1.66	1.51–2.17	0.24
aPTT [sec]	41.5	34.3–46.6	53.4	45.25–79.5	0.30
Leucocytes [/nl]	7.22	4.2–10.9	15.1	7.1–21.2	0.40
CLIF-C AD	53	43–59	63	56–67	0.44
CLIF-C OF	7	7.0–10.0	13	9.0–15.2	0.049*
CLIF-C ACLF	46.5	32.75–49	61	53–67.2	0.30
Outcome	**N**	**%**	**N**	**%**	**P**
5-day rebleeding	3	8.6	10	40.0	< 0.001*
Post-bleeding infection	12	34.2	13	52.0	0.17
Post-bleeding sepsis	2	5.7	10	40.0	0.001*

Continuous data are shown as median and interquartile range, while categorical data as absolute and relative frequencies. P: Results of chi-square test for categorical variables and P_1_: Mann-Whitney U for continuous variables. For brevity purposes, we omitted some variables that had no statistical significance (cause of portal hypertension, presence of HCC, all medication at baseline, FIB4 score, antibiotics prior to or after endoscopy)

*aPTT* activated partial thromboplastin time, *PPI* proton pump inhibitor, *ICU* intensive care unit, *IQR* interquartile range

**Fig. 5:**
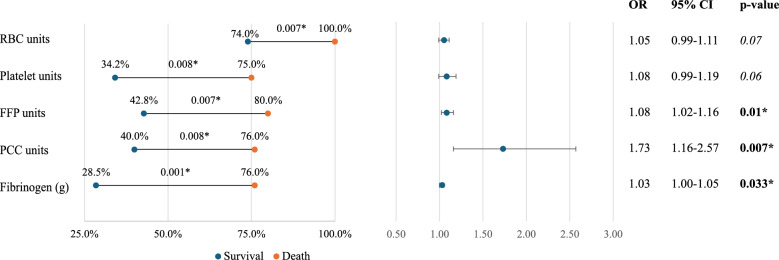
Blood products necessary for the management of EVL-UB. We demonstrate the proportion of patients receiving each blood product and the odds ratios for 6-week mortality per unit increase (per g for fibrinogen) using univariate logistic regression analysis. EVL-UB: endoscopic variceal ligation induced ulcer bleeding, RBC: red blood cell, FFP: fresh frozen plasma, PCC prothrombin complex concentrate

Regarding the transfusion of blood products, patients who died within 6 weeks received more often red blood cell (RBC) units, platelets, fresh frozen plasma (FFP), prothrombin complex concentrate (PCC) units and fibrinogen. In the univariate regression analysis, the administration of FFP, PCC units and fibrinogen were associated with 6-week mortality, while the administration of RBC and platelet units showed a similar trend therefor (Fig. 5). Four thromboembolic events occurred in our cohort; we found no correlation between them and the amount of blood products administered (data not shown). Additionally, patients with 5-day rebleeding received in median 17 RBC units (13.5–30) vs. 4 (2–7) for patients with no rebleeding (*p* < 0.001). Five patients required more than 3 endoscopies due to rebleeding. Overall, patients received a median of 6 RBC units (2.5–14).

Given the number of deaths (events) and the relatively large number of statistically significant results in the univariate analysis, we could not include all significant variables in the multivariate model. Given 25 deaths, we limited multivariate models to 2–3 predictors. We selected those with higher statistical and clinical significance. The most robust model was selected using goodness-of-fit statistics. Post-bleeding sepsis was the strongest predictor of 6-week mortality when analyzing the whole cohort (OR 14.25, 2.1–98.92; *p* = 0.016). 5-day rebleeding was the most significant risk factor for the patients that survived the first 48 h after the EVL-induced ulcer bleeding episode (OR 8.05, 1.66–39.2; *p* = 0.01, Table 3). To complement the primary logistic regression analysis, Cox proportional hazards regression was performed as a sensitivity analysis, accounting for the timing of events over 6-weeks. Five-day rebleeding remained a strong independent predictor of 6-week mortality (HR 4.00; 95% CI 1.33–12.06; *p* = 0.01), while post-bleeding sepsis did not retain significance. It is worth noting that the wide confidence intervals reflect considerable statistical uncertainty in the categorical estimates due to the small sample size and limited number of outcome events.

**Table 3:** Multivariate logistic and cox regression analysis for 6-week mortality after EVL-induced ulcer bleeding. Model A includes 5-day rebleeding. Six patients died within 2 days after initial bleeding. Since we could not assess accurately 5-day rebleeding in those patients, they were excluded from model A (*N* = 54), Model B includes all patients (*N* = 60) but excludes 5-day rebleeding. Akaike Information Criterion: Logistic: Model A 58.27, Model B 62.38 and Cox: Model A: 133.66, Model B: 173.74

Parameter	Model A				Model B			
	OR	95 CI		*P*_1_	OR	95 CI	*P*_2_	
5-day rebleeding	8.05	1.66–39.2		0.01*				
Post-bleeding sepsis	7.27	1.15–45.73		0.04*	14.25	2.1–98.92	0.01*	
Active bleeding					5.41	1.23–23.72	0.01*	
ACLF at the time of EVL-ulcer bleeding					6.58	1.58–27.45	0.04*	
Parameter	**HR**	**95 CI**		**P**_**1**_	**HR**	**95 CI**	**P**_**2**_	
5-day rebleeding	4.00	1.33–12.06		0.01*				
Post-bleeding sepsis	2.01	0.66–6.16		0.22	2.56	1.07–5.99	0.04*	
Active bleeding					2.54	1.08–5.99	0.03*	
ACLF at the time of EVL-ulcer bleeding					3.92	1.25–12.27	0.02*	

*OR* Odds Ratio, *HR* Hazard Ratio

## Discussion

This study provides novel insights into the epidemiology, treatment patterns, and outcomes of EVL-induced ulcer bleeding in real-world practice. We make three principal observations. First, bleeding risk is not uniform across EVL indications. Semi-elective EVLs in acutely decompensated patients carry 45-fold higher odds for this complication than elective prophylactic EVL. Second, multiple endoscopic approaches achieved high primary hemostasis rates. Third, mortality is driven by rebleeding and post-bleeding sepsis rather than endoscopic therapy.

Semi-elective EVL was associated with a significantly higher incidence of ulcer bleeding compared to elective procedures, a subgroup not previously assessed [6–8]. Admission in the ICU at the time of EVL, higher MELD score, and prolonged aPTT emerged as possible risk factors, suggesting that decompensation severity may drive bleeding risk. Prior beta-blocker therapy did not increase the occurrence of this complication, though we could not assess whether beta-blockers were discontinued after ligation. We hypothesize that acutely decompensated cirrhosis is a distinct high-risk setting for EVL, potentially due to a combination of elevated HVPG (hepatic vein pressure gradient) [25], a more fragile and dysregulated hemostatic balance [26], systemic inflammation [27], and impaired mucosal healing [28, 29]. Prospective studies are needed to further characterize risk factors in this distinct clinical context and to determine whether the procedural risk of this complication outweighs the risk of deferring treatment in acutely decompensated patients with high-risk varices.

Repeat EVL and fibrin glue injection were the most commonly used endoscopic treatments, consistent with published data [12]. Multiple modalities achieved high primary hemostasis rates (EVL 83%, fibrin glue 88%, balloon tamponade 75%), comparable to a case series reporting hemostasis rates of 80%, though the authors did not differentiate between endoscopic treatment modalities [6]. Treatment selection was non-randomized and driven by clinical context, therefore no efficacy comparisons were performed. Despite these high hemostasis rates, mortality remained substantial, suggesting that endoscopic treatment success alone does not determine prognosis.

This is further illustrated by the subgroup of conservatively treated patients with clear fibrin-covered ulcers who received no endoscopic treatment. All patients without ACLF survived, whereas 42.9% of the patients with ACLF did not. Furthermore, the overall 6-week mortality rate of 28.5% in this subgroup was 7 times higher than the mortality rate of peptic FIII ulcers (4.7%) [30]. This finding highlights a fundamental difference between peptic ulcer bleeding and EVL-induced ulcer bleeding: in portal hypertension, the bleeding episode itself triggers a cascade of hepatic decompensation, infection, ACLF and multi-organ dysfunction that drives mortality independent of hemostasis.

Consistent with the above findings, hepatic encephalopathy at the time of EVL-induced ulcer bleeding emerged as an independent predictor of 5-day rebleeding, and ACLF showed a trend toward the same direction, with rebleeding rates increasing with ACLF severity. The 5-day rebleeding rate in our cohort (25%) was markedly higher than the 7.7% reported in a prior meta-analysis [10] and the 13–15% rebleeding rate observed in studies on acute variceal bleeding [31, 32]. Additionally, rebleeding occurred earlier at a median of 2 days (0.5–3.5) compared to the previously mentioned meta-analysis, which observed an average of 11.2 ± 3.2 days till rebleeding [10]. These discrepancies likely reflect the higher disease severity of our cohort, as assessed by higher mean MELD scores. The trend toward higher 5-day rebleeding in patients with ACLF is consistent with the existing literature. ACLF has been associated with double risk for rebleeding following variceal hemorrhage [33]. Furthermore, higher ACLF grades have been associated with HVPG > 20 mmHg, suggesting that progressive portal hypertension may represent one of the key mechanisms underlying the increased rebleeding risk observed in more advanced ACLF [34].

Regarding mortality we observed a 6-week mortality rate of 41.7%, which consistent with previous studies ranging from 11% to 63% [7, 10, 12]. By comparison, the 6-week mortality of esophageal variceal bleeding reaches 15–25% [35], but this rate increases when other complications of cirrhosis occur [36, 37]. Multivariate logistic regression identified 5-day rebleeding and post-bleeding sepsis as the strongest independent predictors of 6-week mortality.

The robustness of 5-day rebleeding as a mortality predictor was confirmed in a sensitivity analysis using Cox proportional hazards regression. Post-bleeding sepsis did not retain independent significance in the Cox model likely reflecting two complementary mechanisms: collinearity with 5-day rebleeding, as patients who rebleed are at substantially higher risk of developing sepsis, and the fact that sepsis-related deaths tend to occur later in the clinical course, attenuating the hazard ratio in time-to-event analysis. These findings are consistent with the logistic regression model and further support early rebleeding prevention as a critical target for mortality reduction.

As only 2 patients received TIPS preemptively, we could not assess whether TIPS placement might prevent rebleeding and improve clinical outcomes. In acute variceal bleeding, pre-emptive TIPS placement in high-risk patients reduces rebleeding and improves survival [38, 39], even in patients with ACLF [33, 40] and/or hepatic encephalopathy [41]. Pre-emptive TIPS could theoretically benefit selected high-risk patients with EVL-induced bleeding. In our cohort, 87.5% of TIPS procedures were performed after the first rebleeding episode, representing salvage therapy in deteriorating patients rather than pre-emptive intervention. Nearly all TIPS patients developed infections, and the majority had advanced ACLF, thus confounding outcome interpretation. Therefore, the benefit of pre-emptive TIPS placement in this clinical setting requires further prospective investigation and risk stratification.

Infectious complications represented a major source of morbidity in this cohort, with post-bleeding sepsis emerging as an independent predictor of 6-week mortality in both logistic regression models. Despite antibiotic prophylaxis in 83% of patients, 41.7% developed an infection and 20% sepsis, mirroring observations in acute variceal bleeding. Pneumonia was the most common infection in the post-bleeding phase. This implies that antibiotic prophylaxis may not fully mitigate infection risk in this severely ill population with compromised immune function and frequent aspiration events [42, 43], highlighting the need for additional sepsis prevention and treatment strategies in this setting.

Beyond endoscopic interventions, the role of PPIs in EVL-induced ulcer bleeding remains unclear. While PPIs reduce ligation-induced ulcer size, they do not prevent EVL-induced ulcer bleeding [19]. Published data on PPIs for the management of EVL-induced ulcer bleeds are conflicting [6, 18]. In our cohort, PPI administration did not affect 5-day rebleeding rates, and the observed higher mortality with high-dose PPI likely represents confounding by indication, as ICU patients, who received high-dose PPI more frequently, had higher baseline morbidity. Given the retrospective design and small sample size of our cohort, these findings should be considered exploratory. The role of standard-dose PPIs in EVL-induced ulcer bleeding requires further prospective evaluation.

Finally, high blood product requirements were associated with 6-week mortality and 5-day rebleeding, primarily reflecting disease severity and hemorrhagic burden. Our observational design and lack of data on adherence to restrictive transfusion protocols preclude causal inference. Nevertheless, given that liberal transfusion strategies have been associated with exacerbation of portal hypertension and worse outcomes in the variceal bleeding setting [44], adherence to restrictive transfusion thresholds appears prudent in this population as well.

## Limitations of the study

This study has important limitations inherent to its retrospective, single center, observational design. Most importantly, we cannot compare the effectiveness of endoscopic treatment due to selection bias. The treatment selection was non-randomized, clinician-dependent, and influenced by bleeding severity and patient acuity. The absence of standardized treatment protocol has created heterogeneity, but also reflects real-world practice where treatment must be individualized. EVLs were performed by 15 endoscopists with varying levels of expertise across two sites, which may have introduced variability in procedural technique and outcomes. Additionally, the retrospective design precluded systematic collection of all clinically relevant parameters, limiting the comprehensiveness of the risk factor analysis. The small sample size and limited number of outcome events constrained the multivariate analysis, precluding inclusion of all candidate variables and resulting in wide confidence intervals that reflect the inherent uncertainty of estimates derived from small cohorts. As a tertiary referral center with high rates of ACLF, our population may not represent community practice, and hemostasis rates achieved by our experienced endoscopists may not be reproducible in lower-volume settings. Therefore, generalizability to less specialized centers remains uncertain. Despite these limitations our cohort represents one of the largest single-center cohorts for this rare complication and provides important descriptive data on real-world treatment strategies and outcomes. As with all observational studies, the associations identified in this analysis do not imply causality and should be interpreted accordingly.

## Conclusions

In conclusion, EVL-induced ulcer bleeding is rare after elective EVL (0.4%) but common after semi-elective EVL in acutely decompensated patients (16%). Endoscopic hemostasis is often achievable with various techniques, but mortality remains high (41.7%), driven by early rebleeding and post-bleeding sepsis, rather than endoscopic therapy. This pragmatic observation may be reassuring for endoscopists who must make rapid treatment decisions in critically ill patients and choose endoscopic treatment modality based on endoscopic presentation, local expertise, anatomic factors, and technical feasibility. For patients with clean fibrin-covered ulcers and no ACLF, conservative management may be safe. Multicenter prospective studies are needed to identify which patients should avoid EVL during decompensation, to determine the optimal role of pre-emptive TIPS and to evaluate strategies for preventing post-bleeding sepsis.

## Supplementary Information

Supplementary Material 1.

## Data Availability

The datasets generated and analysed during the current study are not publicly available, as they contain clinical data subject to patient confidentiality obligations and German and European data protection law (DSGVO/GDPR). Given the limited sample size of the study cohort, full anonymisation of individual patient data cannot be reliably ensured, and public deposition carries a meaningful risk of patient re-identification. The data do not include any direct or indirect identifiers in the manuscript. As stated above, this study was conducted as a retrospective analysis of existing clinical and registry data. Therefore, the requirement for individual informed consent was waived by the Ethics Committee of Charité – Universitätsmedizin Berlin (approval reference: EA1/146/22). The dataset may nonetheless be made available from the corresponding author upon reasonable request, subject to institutional approval by the Charité – Universitätsmedizin Berlin data protection authority.

## References

[1] Gulamhusein AF, Kamath PS. The epidemiology and pathogenesis of gastrointestinal varices. Techniques Gastrointest Endoscopy. 2017;19(2):62–8. 10.1016/j.tgie.2017.03.005.

[2] Gralnek IM, Camus Duboc M, Garcia-Pagan JC, Fuccio L, Karstensen JG, Hucl T, et al. Endoscopic diagnosis and management of esophagogastric variceal hemorrhage: European Society of Gastrointestinal Endoscopy (ESGE) Guideline. Endoscopy. 2022;54(11):1094–120. 10.1055/a-1939-4887. 36174643 10.1055/a-1939-4887

[3] de Franchis R, Bosch J, Garcia-Tsao G, Reiberger T, Ripoll C. Baveno VII - Renewing consensus in portal hypertension. J Hepatol. 2022;76(4):959–74. 10.1016/j.jhep.2021.12.022. 35120736 10.1016/j.jhep.2021.12.022 PMC11090185

[4] Gluud LL, Klingenberg S, Nikolova D, Gluud C. Banding ligation versus beta-blockers as primary prophylaxis in esophageal varices: systematic review of randomized trials. Am J Gastroenterol. 2007;102(12):2842–8. 10.1111/j.1572-0241.2007.01564.x. quiz 1, 9. 18042114 10.1111/j.1572-0241.2007.01564.x

[5] Polski JM, Brunt EM, Saeed ZA. Chronology of histological changes after band ligation of esophageal varices in humans. Endoscopy. 2001;33(5):443–7. 10.1055/s-2001-14259. 11396765 10.1055/s-2001-14259

[6] Tierney A, Toriz BE, Mian S, Brown KE. Interventions and outcomes of treatment of postbanding ulcer hemorrhage after endoscopic band ligation: a single-center case series. Gastrointest Endosc. 2013;77(1):136–e401. 10.1016/j.gie.2012.08.031. 23062759 10.1016/j.gie.2012.08.031

[7] Vanbiervliet G, Giudicelli-Bornard S, Piche T, Berthier F, Gelsi E, Filippi J, et al. Predictive factors of bleeding related to post-banding ulcer following endoscopic variceal ligation in cirrhotic patients: a case-control study. Aliment Pharmacol Ther. 2010;32(2):225–32. 10.1111/j.1365-2036.2010.04331.x. 20412065 10.1111/j.1365-2036.2010.04331.x

[8] Kang SH, Yim HJ, Kim SY, Suh SJ, Hyun JJ, Jung SW, et al. Proton Pump Inhibitor Therapy Is Associated With Reduction of Early Bleeding Risk After Prophylactic Endoscopic Variceal Band Ligation: A Retrospective Cohort Study. Med (Baltim). 2016;95(8):e2903. 10.1097/md.0000000000002903. 10.1097/MD.0000000000002903 PMC4779029 26937932

[9] Jamwal KD, Maiwall R, Sharma MK, Kumar G, Sarin SK. Case Control Study of Post-endoscopic Variceal Ligation Bleeding Ulcers in Severe Liver Disease: Outcomes and Management. J Clin translational Hepatol. 2019;7(1):32–9. 10.14218/jcth.2018.00059. 10.14218/JCTH.2018.00059 PMC6441646 30944817

[10] Giri S, Sundaram S, Jearth V, Bhrugumalla S. Predictors of early bleeding after endoscopic variceal ligation for esophageal varices: a systematic review and meta-analysis. Clin Exp Hepatol. 2022;8(4):267–77. 10.5114/ceh.2022.123096. 36683871 10.5114/ceh.2022.123096 PMC9850299

[11] Chen CW, Kuo CJ, Lee CW, Kuo T, Chiu CT, Lin CJ, et al. Albumin-Bilirubin Grade as a Novel Predictor of the Development and Short-Term Survival of Post-Banding Ulcer Bleeding Following Endoscopic Variceal Ligation in Cirrhotic Patients. Med (Kaunas). 2022;58(12). 10.3390/medicina58121836. 10.3390/medicina58121836 PMC9788267 36557038

[12] de Brito Nunes M, Knecht M, Wiest R, Bosch J, Berzigotti A. Predictors and management of post-banding ulcer bleeding in cirrhosis: A systematic review and meta-analysis. Liver international: official J Int Association Study Liver. 2023;43(8):1644–53. 10.1111/liv.15621. 10.1111/liv.15621 37222256

[13] Kim EK, Sohn JH, Kim TY, Kim BK, Yu YH, Eun CS, et al. [Esophageal sinus formation due to cyanoacrylate injection for esophageal variceal ligation-induced ulcer bleeding in a cirrhotic patient]. Korean J Gastroenterol. 2011;57(3):180–3. 10.4166/kjg.2011.57.3.180. 21519166 10.4166/kjg.2011.57.3.180

[14] Mansilla-Vivar R, Vargas JI, Parra-Blanco A. Endoscopic hemostasis with hemoclips for post-variceal ligation bleeding ulcer. VideoGIE. 2020;5(2):56–7. 10.1016/j.vgie.2019.10.002. 32051909 10.1016/j.vgie.2019.10.002 PMC7003062

[15] Lopimpisuth C, Kerdsirichairat T, Khoshknab MP, Hamilton JP, Ngamruengphong S. Over-the-scope clip as salvage therapy in refractory bleeding from esophageal variceal band ligation-induced ulcer. Endoscopy. 2020;52(1):E33–4. 10.1055/a-0983-8230. 31434152 10.1055/a-0983-8230

[16] Sanglodkar UA, Jothimani D, Rela M. Hemospray for recurrent esophageal band ulcer bleeding. Clin Exp Hepatol. 2018;4(1):46–8. 10.5114/ceh.2018.73668. 29594199 10.5114/ceh.2018.73668 PMC5865909

[17] Choudhary NS, Puri R, Saigal S, Saraf N, Sud R, Soin AS. Innovative Approach of Using Esophageal Stent for Refractory Post-Band Ligation Esophageal Ulcer Bleed Following Living Donor Liver Transplantation. J Clin Exp Hepatol. 2016;6(2):149–50. 10.1016/j.jceh.2016.01.003. 27493462 10.1016/j.jceh.2016.01.003 PMC4963324

[18] Cho E, Jun CH, Cho SB, Park CH, Kim HS, Choi SK, et al. Endoscopic variceal ligation-induced ulcer bleeding: What are the risk factors and treatment strategies? Med (Baltim). 2017;96(24):e7157. 10.1097/md.0000000000007157. 10.1097/MD.0000000000007157 PMC5478333 28614248

[19] Dueñas E, Cachero A, Amador A, Rota R, Salord S, Gornals J, et al. Ulcer bleeding after band ligation of esophageal varices: Risk factors and prognosis. Dig Liver Dis. 2020;52(1):79–83. 10.1016/j.dld.2019.06.019. 31395524 10.1016/j.dld.2019.06.019

[20] Moreau R, Tonon M, Krag A, Angeli P, Berenguer M, Berzigotti A, et al. EASL Clinical Practice Guidelines on acute-on-chronic liver failure. J Hepatol. 2023;79(2):461–91. 10.1016/j.jhep.2023.04.021. 37364789 10.1016/j.jhep.2023.04.021

[21] Jalan R, Pavesi M, Saliba F, Amorós A, Fernandez J, Holland-Fischer P, et al. The CLIF Consortium Acute Decompensation score (CLIF-C ADs) for prognosis of hospitalised cirrhotic patients without acute-on-chronic liver failure. J Hepatol. 2015;62(4):831–40. 10.1016/j.jhep.2014.11.012. 25463539 10.1016/j.jhep.2014.11.012

[22] Singer M, Deutschman CS, Seymour CW, Shankar-Hari M, Annane D, Bauer M, et al. The Third International Consensus Definitions for Sepsis and Septic Shock (Sepsis-3). JAMA. 2016;315(8):801–10. 10.1001/jama.2016.0287. 26903338 10.1001/jama.2016.0287 PMC4968574

[23] Martínez J, Hernández-Gea V, Rodríguez-de-Santiago E, Téllez L, Procopet B, Giráldez Á, et al. Bacterial infections in patients with acute variceal bleeding in the era of antibiotic prophylaxis. J Hepatol. 2021;75(2):342–50. 10.1016/j.jhep.2021.03.026. 33845059 10.1016/j.jhep.2021.03.026

[24] Zabor EC, Reddy CA, Tendulkar RD, Patil S. Logistic Regression in Clinical Studies. Int J Radiat Oncol Biol Phys. 2022;112(2):271–7. 10.1016/j.ijrobp.2021.08.007. 34416341 10.1016/j.ijrobp.2021.08.007

[25] Ripoll C, Groszmann R, Garcia-Tsao G, Grace N, Burroughs A, Planas R, et al. Hepatic venous pressure gradient predicts clinical decompensation in patients with compensated cirrhosis. Gastroenterology. 2007;133(2):481–8. 10.1053/j.gastro.2007.05.024. 17681169 10.1053/j.gastro.2007.05.024

[26] Aiza-Haddad I, Cisneros-Garza LE, Morales-Gutiérrez O, Malé-Velázquez R, Rizo-Robles MT, Alvarado-Reyes R, et al. Guidelines for the management of coagulation disorders in patients with cirrhosis. Rev Gastroenterol Mex (Engl Ed). 2024;89(1):144–62. 10.1016/j.rgmxen.2023.08.008. 38600006 10.1016/j.rgmxen.2023.08.008

[27] Trebicka J, Fernandez J, Papp M, Caraceni P, Laleman W, Gambino C, et al. The PREDICT study uncovers three clinical courses of acutely decompensated cirrhosis that have distinct pathophysiology. J Hepatol. 2020;73(4):842–54. 10.1016/j.jhep.2020.06.013. 32673741 10.1016/j.jhep.2020.06.013

[28] Siringo S, Burroughs AK, Bolondi L, Muia A, Di Febo G, Miglioli M, et al. Peptic ulcer and its course in cirrhosis: an endoscopic and clinical prospective study. J Hepatol. 1995;22(6):633–41. 10.1016/0168-8278(95)80219-3. 7560857 10.1016/0168-8278(95)80219-3

[29] Saad RJ, Chey WD. Peptic ulcer disease in patients with chronic liver disease: looking beyond bugs and drugs. Gastrointest Endosc. 2005;62(3):357–9. 10.1016/j.gie.2005.06.003. 16111951 10.1016/j.gie.2005.06.003

[30] de Groot NL, van Oijen MG, Kessels K, Hemmink M, Weusten BL, Timmer R, et al. Reassessment of the predictive value of the Forrest classification for peptic ulcer rebleeding and mortality: can classification be simplified? Endoscopy. 2014;46(1):46–52. 10.1055/s-0033-1344884. 24218308 10.1055/s-0033-1344884

[31] Bambha K, Kim WR, Pedersen R, Bida JP, Kremers WK, Kamath PS. Predictors of early re-bleeding and mortality after acute variceal haemorrhage in patients with cirrhosis. Gut. 2008;57(6):814–20. 10.1136/gut.2007.137489. 18250126 10.1136/gut.2007.137489

[32] D’Amico G, De Franchis R. Upper digestive bleeding in cirrhosis. Post-therapeutic outcome and prognostic indicators. Hepatology (Baltimore MD). 2003;38(3):599–612. 10.1053/jhep.2003.50385. 12939586 10.1053/jhep.2003.50385

[33] Trebicka J, Gu W, Ibáñez-Samaniego L, Hernández-Gea V, Pitarch C, Garcia E, et al. Rebleeding and mortality risk are increased by ACLF but reduced by pre-emptive TIPS. J Hepatol. 2020;73(5):1082–91. 10.1016/j.jhep.2020.04.024. 32339602 10.1016/j.jhep.2020.04.024

[34] Taru V, Kramer G, Hofer BS, Dominik N, Balcar L, Schneeweiss-Gleixner M, et al. Impact of Underlying Portal Hypertension on Severity and Course of Acute-On-Chronic Liver Failure. Liver Int. 2025;45(10):e70363. 10.1111/liv.70363. 40985691 10.1111/liv.70363 PMC12456108

[35] Reverter E, Tandon P, Augustin S, Turon F, Casu S, Bastiampillai R, et al. A MELD-based model to determine risk of mortality among patients with acute variceal bleeding. Gastroenterology. 2014;146(2):412–e193. 10.1053/j.gastro.2013.10.018. 24148622 10.1053/j.gastro.2013.10.018

[36] D’Amico G, Pasta L, Morabito A, D’Amico M, Caltagirone M, Malizia G, et al. Competing risks and prognostic stages of cirrhosis: a 25-year inception cohort study of 494 patients. Aliment Pharmacol Ther. 2014;39(10):1180–93. 10.1111/apt.12721. 24654740 10.1111/apt.12721

[37] D’Amico G, Morabito A, D’Amico M, Pasta L, Malizia G, Rebora P, et al. Clinical states of cirrhosis and competing risks. J Hepatol. 2018;68(3):563–76. 10.1016/j.jhep.2017.10.020. 29111320 10.1016/j.jhep.2017.10.020

[38] Huang Y, Wang X, Li X, Sun S, Xie Y, Yin X. Comparative efficacy of early TIPS, Non-early TIPS, and Standard treatment in patients with cirrhosis and acute variceal bleeding: a network meta-analysis. Int J Surg (London England). 2024;110(2):1149–58. 10.1097/js9.0000000000000865. 10.1097/JS9.0000000000000865 PMC10871647 37924494

[39] Lv Y, Yang Z, Liu L, Li K, He C, Wang Z, et al. Early TIPS with covered stents versus standard treatment for acute variceal bleeding in patients with advanced cirrhosis: a randomised controlled trial. lancet Gastroenterol Hepatol. 2019;4(8):587–98. 10.1016/s2468-1253(19)30090-1. 31153882 10.1016/S2468-1253(19)30090-1

[40] Kumar R, Kerbert AJC, Sheikh MF, Roth N, Calvao JAF, Mesquita MD, et al. Determinants of mortality in patients with cirrhosis and uncontrolled variceal bleeding. J Hepatol. 2021;74(1):66–79. 10.1016/j.jhep.2020.06.010. 32561318 10.1016/j.jhep.2020.06.010

[41] Rudler M, Hernández-Gea V, Procopet BD, Giráldez A, Amitrano L, Villanueva C, et al. Hepatic encephalopathy is not a contraindication to pre-emptive TIPS in high-risk patients with cirrhosis with variceal bleeding. Gut. 2023;72(4):749–58. 10.1136/gutjnl-2022-326975. 36328772 10.1136/gutjnl-2022-326975

[42] Lee S, Saxinger L, Ma M, Prado V, Fernández J, Kumar D, et al. Bacterial infections in acute variceal hemorrhage despite antibiotics-a multicenter study of predictors and clinical impact. United Eur Gastroenterol J. 2017;5(8):1090–9. 10.1177/2050640617704564. 10.1177/2050640617704564 PMC5721982 29238587

[43] Martínez J, Téllez L, Albillos A. Acute variceal bleeding in patients on primary prophylaxis with nonselective beta-blockers: A poor prognosis factor? Hepatology (Baltimore. Md). 2017;65(5):1774. 10.1002/hep.28979. 10.1002/hep.28979 27943346

[44] Odutayo A, Desborough MJ, Trivella M, Stanley AJ, Dorée C, Collins GS, et al. Restrictive versus liberal blood transfusion for gastrointestinal bleeding: a systematic review and meta-analysis of randomised controlled trials. Lancet Gastroenterol Hepatol. 2017;2(5):354–60. 10.1016/s2468-1253(17)30054-7. 28397699 10.1016/S2468-1253(17)30054-7

