# Supplementary material for: Endoscopic variceal ligation-induced ulcer bleeding: incidence by indication, real-world treatment, and outcomes
Source: BMC Gastroenterol. 2026 Jul 24;26:474. doi: 10.1186/s12876-026-05141-5 (PMC13397640; doi:10.1186/s12876-026-05141-5)
Supplement: Supplementary file 1 — Supplementary Material 1. [file 12876_2026_5141_MOESM1_ESM.docx]

***Case-Control sub-study***

*Bleeding cohort and control group characteristics*

Table 1 demonstrates the characteristics of both groups, including epidemiological data, baseline medications, variceal status and laboratory parameters at baseline EVL. Patients in the bleeding cohort tended to be younger (56.5 vs. 61 years; p=0.07). Moreover, we observed a higher intake of anticoagulants (20.3% vs. 5.4%; p=0.005) and a longer aPTT (43.7 secs. vs. 40.2 secs.; p=0.022) in the bleeding group in comparison to the control group. Patients with EVL-induced ulcer bleeding tended to have a higher rate of ascites at baseline EVL (p=0.07).

*Risk factors for EVL-induced bleeding*

Due to the difference in odds between indications for EVL, we performed a subgroup analysis based on the EVL indication to remove this as a confounding factor. Due to the small number of EVL-induced ulcer bleeds after elective EVL, we analyzed only the semi-elective and emergency EVL groups (see Table 2). The semi-elective EVL subgroup is described in the main article.

In the subgroup of emergency EVL, the presence of ascites was associated with a fourfold increase in odds for EVL-induced ulcer bleeding (OR: 4.12, 95 CI 1.08–15.67). The presence of ascites indicates a higher portal pressure that predisposes to variceal bleeding, and probably also to EVL-induced bleeding. We did not observe this result in the semi-elective group, suggesting that this finding may be cohort-dependent and should be interpreted with caution. Neither the CLIF-C scores nor FIB4 score could predict the occurrence of EVL-induced ulcer bleeding in the emergency setting.

| Parameters | Cohort | | | Control group | | | | P -value | |
| --- | --- | --- | --- | --- | --- | --- | --- | --- | --- |
|  | **N=60** | | | **N=111** | | | |  | |
| Age (median, IQR) | 56.5 | 46–68 | | 61 | 51–68 | | | | 0.07 |
| Males (n, %) | 47 | 78.3 | | 74 | 66.7 | | | | 0.13 |
| Cause of Portal Hypertension | **N** | **%** | | **N** | **%** | | |  | |
| PVT | 5 | 8.3 | | 5 | 4.5 | | | 0.89 | |
| Hepatic causes | 53 | 85.0 | | 104 | 93.7 | | | 0.21 | |
| - ALD | 30 | 50.0 | | 56 | 50.5 | | | 0.87 | |
| - Viral hepatitis | 14 | 23.3 | | 28 | 25.2 | | | 0.83 | |
| - MASLD | 7 | 11.7 | | 14 | 12.6 | | | 0.76 | |
| - Others | 15 | 25.0 | | 25 | 22.5 | | |  | |
| Hepatic cause with PVT | 2 | 3.3 | | 9 | 8.1 | | | 0.33 | |
| Combination of hepatic causes | 11 | 18.3 | | 14 | 12.6 | | | 0.53 | |
| Post-hepatic causes | 2 | 3.3 | | 2 | 1.8 | | | 0.61 | |
| Clinical Status at Baseline | | | | | | | | | |
| HCC | 14 | 23.3 | | 38 | 34.2 | | | 0.78 | |
| TIPS | 3 | 5.0 | | 6 | 5.4 | | | 0.93 | |
| Dialysis | 2 | 3.3 | | 8 | 7.2 | | | 0.49 | |
| Kidney dysfunction | 2 | 3.3 | | 0 | 0.0 | | | 0.89 | |
| ACLF | | | | | | | 0.60 | | |
| - No ACLF | 43 | 71.7 | | 85 | 76.6 | | |  | |
| - ACLF 1 | 9 | 15.0 | | 16 | 14.4 | | |  | |
| - ACLF 2 | 5 | 8.3 | | 6 | 5.4 | | |  | |
| - ACLF 3 | 3 | 5.0 | | 6 | 5.4 | | |  | |
| Hepatic encephalopathy |  |  | |  |  | | | 0.16 | |
| - None | 41 | 68.3 | | 77 | 69.4 | | |  | |
| - Mild-moderate | 17 | 28.3 | | 26 | 23.4 | | |  | |
| - Severe | 2 | 3.3 | | 4 | 3.6 | | |  | |
| Ascites |  | | |  | | | | *0.07* | |
| - None | 7 | 11.7 | | 25 | 22.5 | | |  | |
| - Mild-moderate | 19 | 31.7 | | 34 | 30.6 | | |  | |
| - Severe | 34 | 56.7 | | 50 | 44.5 | | |  | |
| Medications at Baseline EVL | | | | | | | | | |
| Non-selective b-blockers | 26 | 43.3 | | 43 | 38.7 | | | 0.94 | |
| Proton pump inhibitors | 39 | 65.0 | | 70 | 63.1 | | | 0.73 | |
| Anticoagulants | 12 | 20.3 | | 7 | 5.4 | | | 0.005* | |
| Antiplatelets | 9 | 15.3 | | 11 | 10.0 | | | 0.29 | |
| Lactulose | 20 | 33.9 | | 40 | 36.0 | | | 0.73 | |
| Ornithin-Aspartate | 15 | 25.4 | | 27 | 24.3 | | | 0.95 | |
| Rifaximine | 10 | 16.9 | | 16 | 14.4 | | | 0.76 | |
| Diuretics | 42 | 71.2 | | 75 | 67.6 | | | 0.66 | |
| Endoscopy at Baseline EVL | | | | | | | | | |
| I+II Grade varices | 34 | 56.7 | | 75 | 67.6 | | | 0.13 | |
| Red spots on varices | 38 | 63.3 | | 78 | 70.2 | | | 0.45 | |
| Presence of fundic varices | 8 | 13.3 | | 10 | 9.0 | | | 0.36 | |
| No. of ligations (median, IQR) | 5 | | 4-6 | 5 | | 4-6 | | 0.22 | |
| Laboratory Values at Baseline | **Median** | **IQR** | | **Median** | **IQR** | | | **P-value** | |
| Bilirubin [mg/dl] | 2.8 | 1.3–6.1 | | 1.75 | 0.9–5.1 | | | 0.10 | |
| Creatinine [mg/dl] | 1.1 | 0.82–1.61 | | 0.99 | 0.75–1.36 | | | 0.11 | |
| MELD score | 17.5 | 13–22 | | 15 | 11–21 | | | 0.20 | |
| Platelets [/nl] | 108.5 | 67.3–175.8 | | 108 | 68–153.5 | | | 0.63 | |
| ALT [U/l] | 36 | 24–62.5 | | 37 | 22–56 | | | 0.47 | |
| AST [U/l] | 63 | 50–93 | | 57 | 35.5–89.5 | | | 0.09 | |
| FIB4-score | 6.18 | 3.50–9.25 | | 5.1 | 3.75–9.3 | | | 0.54 | |
| Hb [g/dl] | 9.3 | 8.1–10.8 | | 9.8 | 8.3–11.75 | | | 0.20 | |
| INR | 1.44 | 1.24–1.71 | | 1.34 | 1.18–1.66 | | | 0.17 | |
| aPTT [sec] | 43.7 | 36.9–50.0 | | 40.2 | 34.5–46.6 | | | 0.022* | |
| Leucocytes [/nl]^†^ | 6.6 | 4.32–10.8 | | 6.55 | 4.48–9.5 | | | 0.72 | |
| CLIF-C AD score^†^ | 55 | 48–62 | | 53 | 48–59 | | | 0.34 | |
| CLIF-C OF score^‡^ | 9 | 8–13 | | 9 | 8–11 | | | 0.91 | |
| CLIF-C ACLF score^‡^ | 56 | 47–61 | | 50 | 43–58 | | | 0.60 | |

**Table 1**. Patient characteristics at baseline EVL in the bleeding cohort and control group. Continuous data are shown as median and interquartile range, while categorical data are shown as absolute and relative frequencies. † CLIF-AD Score and leucocytes were not documented in cases of elective EVL. ‡ CLIF-OF and CLIF ACLF were documented only in cases of ACLF. Missing data: 1.8% for hepatic encephalopathy and ascites in the control group as well as 1.7% and 0.9% for medications at baseline in the bleeding cohort and control group respectively. ALD: Alcohol-associated liver disease, MASLD: Metabolic dysfunction-associated steatotic liver disease, PVT: portal vein thrombosis, aPTT: activated partial thromboplastin time, FIB4: fibrosis 4 score, IQR: interquartile range

| Parameter | Semi elective EVL | | | | | Emergency EVL | | | | |
| --- | --- | --- | --- | --- | --- | --- | --- | --- | --- | --- |
|  | **Cases** | | **Controls** | | **P** | **Cases** | | **Controls** | | **P** |
|  | **N=28** | | **N=52** | |  | **N=27** | | **N=50** | |  |
| Age | 57.5 | 51-68 | 61.5 56–67 | | 0.20 | 54 44–68 | | 59.5 48–71 | | 0.42 |
|  | N | % | N | % |  | N | % | N | % |  |
| Males | 22 | 78 | 32 | 61.5 | 0.12 | 20 | 74.5 | 35 | 70.0 | 0.70 |
| Clinical Characteristics | | | | | | | | | | |
| Anticoagulants | 5 | 17.9 | 3 | 5.8 | 0.12 | 5 | 18.5 | 4 | 8.0 | 0.26 |
| B-blockers | 12 | 42.9 | 30 | 57.7 | 0.21 | 16 | 59,3 | 24 | 48,0 | 0.29 |
| Non-sel. b-blockers | 10 | 35.7 | 25 | 48.1 | 0.29 | 15 | 55,6 | 22 | 44.0 | 0.33 |
| HCC | 10 | 35.7 | 16 | 30.8 | 0.65 | 11 | 40.7 | 19 | 38.0 | 0.81 |
| TIPS | 2 | 7.1 | 5 | 9.6 | 0.71 | 0 | 0.0 | 1 | 2.0 | 1.00 |
| Dialysis | 1 | 3.6 | 1 | 1.9 | - | 1 | 3.7 | 7 | 14.0 | 0.25 |
| ACLF |  |  |  |  | 0.24 |  |  |  |  | 0.69 |
| - No ACLF | 20 | 71.4 | 43 | 82.7 |  | 18 | 66.7 | 31 | 62.0 |  |
| - ACLF 1 | 6 | 21.4 | 9 | 17.3 |  | 3 | 11.1 | 7 | 14.0 |  |
| - ACLF 2 | 1 | 3.6 | - | - |  | 4 | 14.8 | 6 | 12.0 |  |
| - ACLF 3 | 1 | 3.6 | - | - |  | 2 | 7.4 | 6 | 12.0 |  |
| Kidney dysfunction | 2 | 7.1 | 0 | 0.0 |  | 0 | 0.0 | 0 | 0.0 |  |
| Hepatic encephalopathy 0.56 0.44 | | | | | | | | | | |
| - None | 19 | 67.9 | 37 | 71.2 |  | 17 | 63.0 | 35 | 70.0 |  |
| - Mild-moderate | 9 | 32.1 | 15 | 28.8 |  | 9 | 33.3 | 11 | 22.0 |  |
| - Severe | 1 | 3.6 | - | - |  | 1 | 3.7 | 4 | 8.0 |  |
| Ascites 0.48 0.03* | | | | | | | | | | |
| - None | 2 | 7.1 | 5 | 9.6 |  | 3 | 11.1 | 18 | 36.0 |  |
| - Mild-moderate | 6 | 21.4 | 15 | 28.8 |  | 13 | 48.1 | 14 | 28.0 |  |
| - Severe | 20 | 71.4 | 32 | 61.5 |  | 11 | 40.7 | 18 | 36.0 |  |
| ICU at Baseline | 4 | 14.3 | 0 | 0.0 | 0.01* | 12 | 44.4 | 28 | 56.0 | 0.33 |
| Laboratory values | **Median** | **IQR** | **Median** | **IQR** |  | **Median** | **IQR** | **Median** | **IQR** |  |
| MELD | 19.5 | 14–23 | 16 | 12–20 | *0.09* | 17 | 13–22 | 16.5 | 11–24 | 0.68 |
| Platelets [/nl] | 103 | 72–169 | 111 | 72–154 | 0.97 | 113 | 64–176 | 110.5 | 54–175 | 0.59 |
| INR | 1.42 | 1.25–1.65 | 1.32 | 1.22–1.60 | 0.22 | 1.5 | 1.29–1.75 | 1.44 | 1.15–1.89 | 0.53 |
| Hb [g/dl] | 10.2 | 8.9–10.9 | 10.7 | 8.8–12.2 | 016 | 8.3 | 7.7–9.7 | 8.6 | 6.9–10.1 | 0.46 |
| aPTT [sec] | 43.7 | 40.7–49.8 | 42.3 | 35.4–46.8 | *0.07* | 43.9 | 34.9–58.8 | 39.7 | 33.1–48.2 | 0.24 |
| CLIF-C AD | 55 | 48–61 | 52 | 44–58 | 0.22 | 55 | 49–68 | 55.5 | 48–63 | 0.63 |
| CLIF-C OF | 8.5 | 7–9.5 | 8 | 8–9 | 0.68 | 12 | 10–14 | 11 | 9–14 | 0.75 |
| CLIF-C ACLF | 46.5 | 43–51 | 44 | 42–49 | 0.50 | 60 | 53–62 | 52 | 48–61 | 0.34 |

**Table 2.** Patient characteristics at baseline EVL in the cohort and control group based on EVL indication. Continuous data are shown as median and interquartile range, while categorical data as absolute and relative frequencies. For brevity purposes, we omitted the following parameters with no statistically significant differences: Medication at baseline (proton pump inhibitors, antiplatelets, rifaximine, lactulose, ornithine-aspartate, and diuretics), lab values at baseline (total bilirubin, sodium, creatinine, AST, ALT, leucocytes), cause of portal hypertension. aPTT: activated partial thromboplastin time, MELD: model of end-stage liver disease.

| **Cause of death** | **N** | **%** |
| --- | --- | --- |
| Cardiogenic shock | 1 | 4 |
| Brain Hemorrhage | 2 | 8 |
| ACLF | 13 | 52 |
| Septic shock | 7 | 28 |
| Bleeding from another source | 1 | 4 |
| Combination hypovolemic/septic shock | 1 | 4 |

**Table 3**. Cause of 6-week mortality. ACLF: acute on chronic liver failure
